# False-positive Bioline TB Ag MPT64 result from MGIT bottles inoculated with *Staphylococcus aureus*

**DOI:** 10.1128/jcm.01711-25

**Published:** 2026-02-10

**Authors:** Matthew Kochan, Valentina Russell, Heather J. Adam, James A. Karlowsky, Anissa Brahami, Andrew Walkty

**Affiliations:** 1 Department of Medical Microbiology and Infectious Diseases, Max Rady College of Medicine, University of Manitoba 8664, Winnipeg, Manitoba, Canada; 2 Shared Health 366539, Winnipeg, Manitoba, Canada; 3 Section of Infectious Diseases, Department of Internal Medicine, Max Rady College of Medicine, University of Manitoba 8664, Winnipeg, Manitoba, Canada; University of Western Australia, Perth, Australia

**Keywords:** MPT64, *Mycobacterium tuberculosis*, *Staphylococcus aureus*

## LETTER

The ability to rapidly identify acid-fast bacilli (AFB) recovered on culture as a member of the *Mycobacterium tuberculosis* complex (MTBC) is an important step in diagnostic mycobacteriology. The Bioline TB Ag MPT64 test (Abbott Diagnostics Korea Inc., Republic of Korea) is an immunochromatographic assay that detects MPT64, an antigen associated with MTBC, using mouse monoclonal antibodies (1, 2). This test can differentiate MTBC from non-tuberculous mycobacteria and may be performed on specimens obtained from a liquid culture (e.g., 100 µL from a BBL MGIT bottle [Becton, Dickinson and Company, Sparks, MD, USA]) or colonies growing on solid media that are emulsified in an extraction buffer (2).

We observed an unexpected false-positive MPT64 antigen result associated with *Staphylococcus aureus* growth in a clinical specimen, which prompted further investigation. Initially, gram-positive cocci and AFB were seen on direct examination of fluid obtained from aspiration of an ischiorectal abscess. *S. aureus* was recovered on aerobic culture. A MGIT bottle inoculated with an untreated aliquot of the specimen was positive after 1 day of incubation and grew *S. aureus*. However, the Kinyoun stain of the MGIT broth showed AFB, prompting the laboratory to perform an MPT64 antigen test, which was positive. A molecular test on the original specimen using the Xpert MTB/RIF Ultra assay (Cepheid, Sunnyvale, CA, USA) was negative, leading us to suspect a false-positive MPT64 antigen result. The AFB that led to the initial MPT64 test was later identified as *Lawsonella clevelandensi*s (isolate recovered on anaerobic bacterial culture), a partially acid-fast anaerobe previously found in human abscesses (3).

We found one published reference that suggested *S. aureus* may cause a false-positive MPT64 antigen test (4) and sought to investigate whether this occurred in our case. In order to simulate the conditions that led to our positive result, one loop from a single colony of a pure *S. aureus* subculture grown on blood agar from the same clinical specimen was inoculated in a MGIT bottle to which the MGIT 960 growth supplement and MGIT PANTA antibiotic mixture were added. After 1 day of incubation, the bottle was positive. An MPT64 antigen test performed on an aliquot of liquid from this bottle demonstrated a weakly positive result.

**Fig 1:**
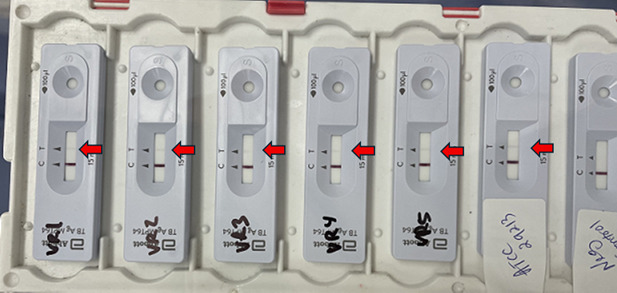
Bioline TB Ag MPT64 test results for five clinical strains of *S. aureus* and the *S. aureus* ATCC 29213 control strain. Testing was performed on an aliquot of specimen obtained from inoculated MGIT bottles. All *S. aureus* isolates gave a positive result (weak band, indicated with a red arrow). Testing performed on a specimen from an uninoculated MGIT bottle (far right) gave a negative result, as expected. Testing results for an additional clinical *S. aureus* isolate (positive) and *S. epidermidis* isolate (negative) are not shown here.

We repeated this procedure using five randomly selected *S. aureus* clinical isolates (two methicillin-resistant isolates and three methicillin-susceptible isolates), *S. aureus* ATCC 29213*,* one *Staphylococcus epidermidis* clinical isolate, and one uninoculated MGIT bottle as a negative control. All six *S*. *aureus* isolates were weakly positive by the MPT64 antigen test (Fig. 1, red arrows), and the *S. epidermidis* was negative. MPT64 tests from two different lot numbers yielded identical results. Interestingly, when we emulsified several colonies of *S. aureus* ATCC 29213 in the extraction buffer (not needed for testing of liquid specimen) and then performed the MPT64 antigen test, we obtained a negative result. It seems that the false-positive results we observed depend on the method in which the test is performed.

The manufacturer reports 98.6% sensitivity and 100% specificity for the MPT64 Ag test for MTBC identification. Kumar et al. evaluated the specificity of the assay using 12 bacterial isolates, including *S. aureus*, and found no false-positive results (5). However, all isolates were tested by emulsification in the extraction buffer rather than inoculation into a MGIT bottle.

These findings have important clinical implications. In high-throughput diagnostic mycobacteriology laboratories, rapid detection of MTBC is relied upon to streamline workflow and deliver timely results. Although not common, a false-positive MPT64 antigen test due to mycobacterial culture contamination with *S. aureus* may lead to unnecessary treatment and patient isolation. The basis for the false-positive results is not clear to us and will require further evaluation. Our study only assessed the Bioline TB Ag MPT64 test, and the applicability of our findings to other brands is not known. It is important for microbiologists to recognize that MPT64 is produced during the active growth of MTBC. As members of the MTBC grow slowly, a MGIT bottle that goes positive after only 1 day of incubation usually represents bacterial contamination, and a positive MPT64 antigen test on a specimen from such a bottle would warrant thorough investigation.
